# Are religious beliefs and practices of Buddhism associated with disability and salivary cortisol in office workers with chronic low back pain?

**DOI:** 10.1186/1471-2474-14-29

**Published:** 2013-01-17

**Authors:** Annop Sooksawat, Prawit Janwantanakul, Tewin Tencomnao, Praneet Pensri

**Affiliations:** 1 Department of Physical Therapy, Faculty of Allied Health Sciences, Chulalongkorn University, Bangkok, Thailand; 2 Center for Excellence in Omics-Nano Medical Technology Development Project, Department of Clinical Chemistry, Faculty of Allied Health Sciences, Chulalongkorn University, Bangkok, Thailand

## Abstract

**Background:**

Low back pain (LBP) is common among office workers. A number of studies have established a relationship between Christianity and physical and mental health outcomes among chronic pain patients. The purpose of this study was to examine the relationship between the religious beliefs and practices of Buddhism and disability and psychological stress in office workers with chronic LBP.

**Methods:**

A cross-sectional survey was conducted with a self-administered questionnaire delivered by hand to 463 office workers with chronic LBP. Saliva samples were collected from a randomly selected sub-sample of respondents (n=96). Disability due to LBP was assessed using the Roland-Morris Disability Questionnaire and psychological stress was assessed based on salivary cortisol. Two hierarchical regression models were built to determine how much variance in disability and psychological stress could be explained by religious beliefs and practices of Buddhism variables after controlling for potential confounder variables.

**Results:**

Only 6% of variance in psychological stress was accounted for by the religious beliefs and practices of Buddhism. Those with high religiousness experienced lower psychological stress. No association between the religious beliefs and practices of Buddhism and disability level was found. Depressive symptoms were attributed to both psychological stress and disability status in our study population.

**Conclusions:**

The findings suggest that, although being religious may improve the psychological condition in workers with chronic LBP, its effect is insufficient to reduce disability due to illness. Further research should examine the role of depression as a mediator of the effect of psychological stress on disability in patients with chronic LBP.

## Background

It is well documented that psychosocial factors significantly influence low back pain (LBP), disability, and persistent symptoms [1,2]. Psychological stress has been shown to exacerbate pain in women with chronic pain, such as fibromyalgia syndrome [3]. Maladaptive pain cognitions, such as pain catastrophizing, cause fear of movement, which in turn contributes to activity avoidance and functional disability [4]. A systematic review suggests that depression is a strong predictor of poor LBP prognosis [5].

Previous studies among Christians showed that religion/spirituality was associated with positive psychological conditions [6]. Baetz and Bowen [7] found that chronic pain and fatigue sufferers who were both religious and spiritual were more likely to have better psychological well-being and use positive coping strategies than non-religious and non-spiritual sufferers. Abraido-Lanza et al. [8] reported a positive association between religious coping and psychological well-being in 200 patients with arthritis. Also, Baetz et al. [9] surveyed 70,884 Canadians and found that those who frequently attended worship services had lower levels of depressive symptoms.

Buddhism is one of the major world religions with most Buddhists living in Asia, particularly in the East and South-East Asia regions. According to the National Statistical Office, 94.2% of the Thai population is Buddhist [10]. Buddhism is a system of teaching aiming to eradicate the ultimate problem of mental suffering in life. Its teaching emphasizes the use of one’s own wisdom to attain the objective truth of nature and completely eliminate the origin of mental distress so that the mind can be released once and for all from suffering. Buddhism preaches that nature is a causally and conditionally interdependent system of phenomena, including the mind and body of man [11]. Mindfulness and meditation, which is the practice of paying attention, on purpose, moment-to-moment, in a way that is non-judgmental and non-reactive [12], are fundamentally parts of Buddhism. Several studies indicated a positive effect of mindfulness on pain catastrophizing, pain-related fear, hyper-vigilance, pain-related anxiety and, physical and psychological functioning [13-15]. Significant improvements in pain intensity, pain acceptance, psychological well-being and physical function have been reported in chronic pain patients following mindfulness-based treatments [12,16]. The practice of continuous mindfulness has been proposed to facilitate the acquisition of adaptive thought and emotion-regulation skills, which reduces psychological inflexibility and expands behavioral repertoires to provide patients with more healthy options to deal with their own problems [13]. In addition, mindfulness enhances physical self-monitoring and body awareness, possibly leading to improved body mechanics and self-care [12].

To date, there has been no study on the effect of religious beliefs and practices of Buddhism on physical and mental health outcomes in chronic musculoskeletal patients, whose physical health is closely related to their psychosocial condition, such as chronic LBP patients. The purpose of this study was to examine the association between the religious beliefs and practices of Buddhism and disability and psychological stress in office workers with chronic LBP. Psychological stress was measured by salivary cortisol, which is routinely used as a biomarker of psychological stress and related mental or physical diseases. Salivary cortisol levels are considered a reliable measure of hypothalamus-pituitary-adrenal (HPA) axis adaptation to stress [17]. Evidence suggests that the HPA axis activity may play a role in the association between psychological variables and chronicity of pain [18]. It was hypothesized that the religious beliefs and practices of Buddhism would be associated with lower levels of disability and psychological stress in this group.

## Methods

A cross-sectional study was conducted on office workers, who were defined as those working in an office environment with their main tasks involving using a computer, participating in meetings, giving presentations, reading, and phoning [19]. Office workers were included if they were Buddhist, aged between 18–60 years and had concurrent chronic LBP (i.e. LBP ≥3 months duration either continuously or intermittently such that pain was experienced at least once per week [20]). The area of LBP was defined according to the standardized Nordic questionnaire [21]. Subjects were excluded if they reported pregnancy or had a history of spinal surgery, trauma, or accidents. Subjects who had been diagnosed with congenital anomaly of the spine, rheumatoid arthritis, infection of the spine and discs, ankylosing spondylitis, lumbar spondylolisthesis, lumbar spondylosis, tumor, systemic lupus erythymatosus, or osteoporosis were also excluded from the study. Inclusion and exclusion criteria were determined by using a self-reported questionnaire.

An invitation letter and information about the study were sent to a convenience sample of office workers in 11 workplaces in Bangkok, Prachinburi and Singburi provinces in Thailand. The enterprises participating in this study were those in public transportation, infrastructure, energy, healthcare, insurance as well as the municipal office and ministerial head office. Those who expressed interest and who were eligible were invited to participate in the study. A self-administered questionnaire was then distributed to each eligible worker by hand and the researcher returned to collect the completed questionnaire 45 minutes later. In addition, saliva samples were collected from a sub-sample of respondents, who were randomly selected using a computer-generated random allocation sequence. Written informed consent was obtained from all participants and the study was approved by the University Human Ethics Committee.

### Religious beliefs and practices of Buddhism questionnaire

The religious beliefs and practices of Buddhism questionnaire is a reliably validated instrument developed by a team of Thai researchers [22]. The questionnaire consisted of 30 questions divided into three subscales: 10 items on belief in the Buddhist teachings, 10 items on Buddhist practice, and 10 items on the Buddhist lifestyle. Respondents are asked to what extent these statements are true for them. Items are scored on a 6-point Likert scale with responses ranging from 1 (absolutely untrue) to 6 (absolutely true). The total score of the test ranges from 30 to 180, with higher scores indicating more spirituality or religiousness (Additional file 1).

The 10 items on belief in Buddhist teachings (Cronbach’s alpha coefficient = .83; Construct validity using the known group technique *t*-test = 13.29, p < .001; Discrimination t-ratio = 5.25 to 11.08) assess an individual’s belief in the three basic teachings of Buddhism as truth, namely 1) belief in the three sources of religious dependence, i.e. the Buddha, His teachings, and the Buddhist monk, 2) belief in the law of cause and effect, heaven and hell, and the cycle of birth and death, and 3) belief in Nirvana or the ultimate goal which can be achieved by a human being.

The 10 items on Buddhist practice (Cronbach’s alpha coefficient = .78; Construct validity *t*-test = 6.02, p < .01; Discrimination t-ratio = 3.48 to 10.48) measure an individual’s action or restraint as relates to being a good Buddhist, namely 1) the act of giving, which includes donations, forgiving and delivering the Buddha’s teachings to others, 2) the Five Precepts which are the sins of commission by words and deeds, and 3) praying and meditating.

The 10 items on Buddhist lifestyle (Cronbach’s alpha coefficient = .85; Discrimination t-ratio = 6.41 to 10.87) assess the extent to which an individual performs the activities of everyday life consonant with Buddhism principles.

### Fear-avoidance beliefs questionnaire

The Fear-Avoidance Beliefs Questionnaire (FABQ) Thai version is a 16-item instrument containing two subscales: 7 items on fear avoidance beliefs about work and 4 items on fear avoidance beliefs about physical activity. Items are scored on a 7-point Likert scale with responses ranging from 0–6 (completely disagree to completely agree). The total score of the FABQ work scale ranges from 0 to 42 and the total score of the FABQ physical activities scale ranges from 0 to 24. Higher scores indicate higher fear avoidance attitudes [23].

### Roland-morris disability questionnaire

Disability level associated with LBP was assessed using the Roland-Morris Disability Questionnaire (RDQ) Thai version, which contains 24 yes/no items. Patients are asked whether the statements apply to them that day (the last 24 hours). The RDQ score is calculated by adding up the number of “yes” items, ranging from 0 to 24, with higher scores indicating more severe disability [24].

### General information and confounder questionnaire

This questionnaire comprised four sections designed to gather data on individual, work-related, and psychosocial factors as well as LBP characteristics. Individual factors included gender, age, height, body weight, educational level, marital status, frequency and duration of weekly exercise sessions, and smoking habits. Respondents were asked if they thought their work was physically demanding and whether they received work compensation due to LBP. Information about expectations of treatment or recovery was also sought.

Psychosocial factors were measured using the General Health Questionnaire (GHQ-28) Thai version, which is a 28-item measure of emotional distress in medical settings. The questions are grouped into four areas: somatic symptoms, anxiety and insomnia, social dysfunction, and severe depression. Each area has 7 questions with a score of 0 or 1. The total score for each area ranges from 0 (better or same as usual symptoms for all questions) to 7 (worse or much worse than usual symptoms for all questions). A combination of scores from four areas with a score of 6 or more indicates a case of psychological distress [25].

Regarding LBP, subjects were asked about the duration of LBP, pain intensity using a numerical pain scale, and radiation of pain in the past 4 weeks.

### Salivary cortisol

An individual’s psychological stress was assessed based on salivary cortisol. Literature suggests that the integrated volume of cortisol released over the waking period is positively related to general life stress [26]. Participants received written and oral information about sampling details. The salivary sample was collected on the Wednesday of the week to reduce day-to-day variation in salivary cortisol by using a salivary sample collection set (IBL-America, Minneapolis, MN, USA). Participants collected two saliva samples: at awakening and 30 min after awakening. Participants were told not to brush their teeth and to refrain from eating and drinking before the end of sampling time. Participants were asked to abstain from food, alcohol, caffeine products, juice, and certain medicines (prednisone, dexamethasone, steroids, adrenergic agonist and antagonist) for at least 3 hours prior to saliva collection. Participants were also asked not to participate in any vigorous activity within 24 hours prior to sample collection. A salivary sample was not collected during a menstruation period. The salivary samples were stored in a −20°C refrigerator until assayed.

The free cortisol concentration in saliva was measured using an enzyme-linked immunosorbent assay (ELISA) kit according to the manufacturer’s instructions (IBL-America). All samples were assayed in duplicate and the average was used in analysis. To quantify the cortisol awakening response of each subject, the “area under the curve with respect to the ground” (AUC_G_) was calculated using the formula outlined by Pruessner et al. [27]. In this study, the two cortisol concentration measurements (i.e. at awakening and 30 min after awakening) represented M_1_ and M_2_ and time interval between the measurements was 30 min. Thus, the total AUC_G_ can be calculated as: (M1+M2)/2 × 30.

Before data collection, the test-retest reliability of data from all questionnaires was assessed using 32 office workers. Each subject was tested twice on 2 separate days with a week lapse between the measurements. The intraclass correlation coefficient [ICC (1,1)] was used for continuous data. Kendall’s tau-b and Phi were calculated for ordinal and nominal data, respectively.

### Statistical analyses

Descriptive statistics were calculated for all variables. General characteristics of subjects in the main study (n=463) and a randomly selected sub-sample of office workers (n=96) were compared using the independent *t*-test and Chi-square for continuous and nominal/ordinal data, respectively.

Two separate hierarchical regression analyses were conducted to determine how much variance in the RDQ score and AUC_G_ could be explained by the religious beliefs and practices of Buddhism variables after controlling for potential confounder variables. Correlational analyses were conducted first to examine the relationships among the religious beliefs and practices of Buddhism and each variable. For dichotomous variables, dummy variables were constructed before performing the correlational analysis. The variables chosen for inclusion in the regression analyses were those significantly correlated with the RDQ score and AUC_G_ based on correlational analysis. All variables were entered in the first step and the religious beliefs and practices of Buddhism variable was entered in the second step. All statistical analyses were performed using SPSS statistical software, version 17.0 (SPSS Inc, Chicago, IL, USA). Statistical significance was set at the 5% level.

## Results

### Test-retest reliability

The reliability results demonstrated moderate to good reliability of questionnaire outcomes with the ICC (1,1) scores ranging from 0.75 to 0.92, Kendall’s tau-b ranging from 0.76 to 0.87 and Phi ranging from 0.85 to 1.00.

### Subjects’ characteristics

There were 2,890 office workers who were approached to participate in the study with 2,250 accepting the invitation (a response rate of 77.8%). Of 2,250 workers, 519 were eligible, but only 463 agreed to participate in this study. They were asked to complete the self-administered questionnaire. Of the 463 workers, 102 were randomly selected but only 96 agreed to collecting their saliva samples (a response rate of 94.1%). Table 1 presents the demographic, LBP, health outcomes, and the Buddhist characteristics of the religious beliefs and practices of the study population. A small number of office workers agreed that their job was physically demanding (21.4%) and received work compensation due to their LBP (15.8%). About 52.1% agreed or strongly agreed that their LBP would completely resolve itself. Mean cortisol levels at awakening for those with higher and lower/equal to the mean total scores of the religious beliefs and practices of Buddhism questionnaire (132.8) were 5.41 and 6.65 μg/dL, respectively. Mean cortisol levels at 30 min after awakening for those with higher and lower/equal to mean total scores than the mean total scores of the religious beliefs and practices of Buddhism questionnaire (132.8) were 7.19 and 8.57 μg/dL, respectively and 30 min after awakening were divided into two groups based on mean total score derived from.

**Table 1:** Demographic, low back pain, health outcomes, and the Buddhist characteristics of the religious beliefs and practices of participating office workers with chronic low back pain

**Characteristics**	**(n=463)**		**(n=96)**		
	**N (%)**	**Mean (SD)**	**N (%)**	**Mean (SD)**	**P-value**
*Demographic characteristics*					
Age		38.5 (10.0)		36.5 (9.1)	0.55
Gender					0.69
Male	113 (24.4)		32 (33.3)		
Female	350 (75.6)		64 (66.7)		
Body mass index (kg/m^2^)		23.1 (3.9)		23.8 (4.5)	0.16
Education					0.42
Lower than Bachelor’s degree	80 (17.3)		14 (14.5)		
Bachelor’s degree	298 (64.4)		59 (61.5)		
Higher than Bachelor’s degree	85 (18.3)		23 (24.0)		
Exercise frequency in the past 12 months					0.34
Never	123 (26.6)		31 (32.3)		
Occasionally	288 (62.2)		52 (54.2)		
Regularly	52 (11.2)		13 (13.5)		
*Low back pain characteristics*					
Duration of low back pain (months)		28.3 (36.9)		27.8 (36.9)	0.89
Pain intensity in the past 4 weeks using NPS		4.3 (1.8)		4.3 (1.8)	0.87
Radiation of pain in the past 4 weeks					0.74
Yes	137 (39.6)		30 (31.2)		
No	326 (70.4)		66 (68.8)		
*Health outcomes*					
RDQ-24		4.8 (3.8)		5.1 (3.6)	0.40
FABQ work subscale		17.8 (7.8)		16.7 (7.6)	0.21
FABQ physical activity subscale		14.5 (4.5)		14.6 (4.4)	0.72
GHQ-28		4.6 (5.0)		5.0 (5.5)	0.47
Anxiety subscale		1.3 (2.0)		1.4 (2.2)	0.77
Depression subscale		0.2 (0.8)		0.4 (1.0)	0.13
AUC_G_ (cortisol) (μg.min/dL)		-		208.2 (79.8)	-
Cortisol level at awakening		-		6.02 (2.5)	
Cortisol level at 30 min after awakening		-		7.87 (3.4)	
*Religious beliefs and practices of Buddhism*					
Total score		135.1 (14.9)		132.8 (15.1)	0.17
Beliefs in Buddhist teaching subscale		43.5 (6.1)		42.0 (6.1)	0.05
Buddhist practice subscale		47.6 (6.3)		46.4 (6.9)	0.09
Buddhist lifestyle subscale		43.9 (6.2)		43.3 (6.3)	0.63

NPS, numerical pain scale; RDQ-24, the Roland-Morris Disability Questionnaire; FABQ, Fear-Avoidance Beliefs Questionnaire; GHQ-28, the General Health Questionnaire; AUC_G_, area under the curve with respect to ground.

### Association between disability and the religious beliefs and practices of Buddhism (n=463)

A model was constructed to establish the association between disability and the religious beliefs and practices of Buddhism (Table 2). When correlational analysis was used, LBP duration, pain intensity in the past 4 weeks, radiation of pain in the past 4 weeks, expectation of treatment or recovery, anxiety, depression, FABQ physical activity and FABQ work subscale were significantly correlated with the RDQ score. The final model explained 27% of the total variance in disability. Twenty-seven percent of variance in disability was accounted for by LBP duration, pain intensity in the past 4 weeks, radiation of pain in the past 4 weeks, depression, FABQ physical activity score, and FABQ work score. No further variance in disability was explained by the religious beliefs and practices of Buddhism after controlling for confounder variables.

**Table 2:** Hierarchical regression analysis predicting disability due to low back pain

		***R***^***2***^	***R***^***2***^**increment for block**	**Standard β**	***F *increment**
Step 1	Potential confounders for disability	.27			21.86***
	LBP duration			.16***	
	Pain intensity in the past 4 weeks			.20***	
	Radiation of pain in the past 4 weeks			.14**	
	Expectation of treatment or recovery			-.07	
	Anxiety			.06	
	Depression			.16**	
	FABQ physical activity subscale			.09*	
	FABQ work subscale			.14**	
Step 2	Religious beliefs and practices of Buddhism	.27	.00		0.51
	Total scale			.02	

Overall model *R*^*2*^ = .279 (*F* = 19.46, *P* = .000).

Betas are standardized. *P < .05; **P < .01; ***P < .001.

LBP, low back pain; FABQ, Fear-Avoidance Beliefs Questionnaire.

### Association between psychological stress and the religious beliefs and practices of Buddhism (n=96)

A model was constructed to establish the association between psychological stress and the religious beliefs and practices of Buddhism (Table 3). When correlational analysis was used, gender, age, education and depression were significantly correlated with AUC_G_. The final model explained 25% of the total variance in psychological stress. Nineteen percent of variance in psychological stress was accounted for by gender, age, education, and depression. The religious beliefs and practices of Buddhism accounted for an additional and significant 6% of variance in psychological stress, after controlling for confounder variables.

**Table 3:** Hierarchical regression analysis predicting physiological stress

		***R***^***2***^	***R***^***2***^**increment for block**	**Standard β**	***F *increment**
Step 1	Potential confounders	.19			5.50**
	Gender			.24*	
	Age			.21*	
	Education			.22*	
	Depression			.27**	
Step 2	Religious beliefs and practices of Buddhism	.25	.06**		7.34**
	Total scale			-.25**	

Overall model *R*^*2*^ = .255 (*F* = 6.17, *P* = .000).

Betas are standardized. *P < .05; **P < .01; ***P < .001.

## Discussion

The analysis of the relationship between the religious beliefs and practices of Buddhism and disability and psychological stress in office workers with chronic LBP revealed that the religious beliefs and practices of Buddhism were significantly associated with psychological stress but not with disability. Workers with high religious beliefs and practices of Buddhism had lower psychological stress. The findings confirm the results of previous studies regarding the effect of religious beliefs and practices of Christianity on psychological conditions [7-9]. A number of hypotheses have been proposed to explain the improvement of psychological conditions due to religious and spiritual practices. These hypotheses have focused on the influence of religious and spiritual practices on neural pathways and social aspects as well as the increase in mindfulness. Positive emotions, such as forgiveness, hope, contentment, love, may reduce the arousal in the endocrine and immune systems and the hypothalamus-pituitary-adrenal axis system, which help increase immune competence and restore physiological stability [28]. Religious or spiritual practices provide opportunities for fellowship, involvement in formal social programs, and companionship, thus helping reduce both psychological and physical stressors [29]. Religious or spiritual practices may increase mindfulness, which reduces psychological inflexibility through an improvement in ‘focused attention’ and facilitation of adaptive thought and emotion-regulation skill acquisition [13,14].

However, based on the results of the present study, the effect of religious beliefs and practices of Buddhism on psychological stress was rather subtle (6%). Rippentrop et al. [30] conducted a study in chronic musculoskeletal pain patients with the majority of patients being Christian and found that 12% of variance in mental health status, measured by the SF-36, among the study sample was accounted for by forgiveness, negative religious coping, daily spiritual experiences, religious support, and spiritual/religiousness intensity. Our findings indicate that the application of religious beliefs and practices to the improvement of psychological conditions in the patient population may be limited. However, only a few selected psychological factors were examined in the present and previous studies. Other important psychological factors that relate to the religious beliefs and practices may be identified in future work.

Several factors were found to significantly associate with disability due to LBP, including duration of LBP, pain intensity in the past 4 weeks, radiation of pain in the past 4 weeks, depression, and fear-avoidance beliefs. However, no association between the religious beliefs and practices of Buddhism and disability was found. Johnstone and Yoon [31] found no association between religiousness/spirituality and physical health in 118 individuals with chronic disabilities, including traumatic brain injury, cerebral vascular accidents and spinal cord injury. Rippentrop et al. [30] reported that only 3% of variance in physical health status, measured by the SF-36, was accounted for by private religious practices in chronic musculoskeletal pain patients. The authors hypothesized that patients with poor physical health relied on their faith for comfort and, thus, private religious activity was a result of increasing physical disability. These findings suggest that, although being religious may improve psychological condition, its effect is insufficient to reduce disability due to illness, at least in our sample of office workers with chronic LBP. However, our study population of office workers with chronic LBP had a low disability level (average RDQ scores of 4.8/24), which is in contrast with that of Turner et al. [32] who reported a moderate level of disability (the mean RDQ score = 12.7/24) for those workers submitting work compensation claims for work-related back pain. This discrepancy may be due to the difference in subject characteristics and occupation. In the previous study, the sample was workers who ceased working because of their LBP condition while in the present study the office workers were still engaged in their work. Workers who keep working should have low disability because it would be difficult for them to remain productive at moderate to high disability levels [33]. Also, the present study only recruited office workers while the previous study included workers from different occupations. Office work is sedentary which mainly involves computer use, participation in meetings, giving presentations, reading, and phoning [19]. Only 21% of participating office workers reported that their job was physically demanding. As a result, we hypothesized that office workers were less likely to have moderate to high disability levels because of their limited physical requirements at work. Thus, extrapolation of the results to chronic LBP office workers with moderate to high disability should be undertaken with caution. Different results may emerge with those having moderate to high disability levels. Further research on the effect of religion/spirituality on physical and mental health in those seeking treatment is recommended.

Interestingly, based on hierarchical multiple regression models, we found that depression was only an investigated variable significantly associated with both psychological stress and disability levels in our study population of office workers with chronic LBP. Office workers with high depressive symptoms had high psychological stress and disability levels, which is consistent with the findings from previous studies [34,35]. Nearly 50% of chronic pain patients suffer from serious depression [36]. Depression, which is characterized by low positive affection and loss of self-esteem, potentially decreases the motivation for activity and thus affects productivity and disability [37]. Religion/spirituality, which has a positive effect on mental health, may partly reduce depression and consequently benefit patients’ physical health. Further research is required to investigate the long-term effect of reduced psychological stress on disability level in chronic LBP patients.

The current study has several weak points. First, saliva samples were collected from a randomly selected sub-sample of office workers participating in this study. Nevertheless, their characteristics were very similar to a larger sample of office workers (n = 463) who participated in this study. Also, salivary sampling in field studies relies on the participants themselves to collect their samples. Thus, it is possible that participants did not collect samples precisely when they were instructed to do so. Second, the cross-sectional study design only allows the association between exposure and outcome to be examined. It is not possible to establish a causal relationship between exposure and outcome. Therefore, a prospective study design is required to validate the findings of this study. Third, this study may be susceptible to the “healthy worker effect”. Office workers suffering from musculoskeletal injury due to work may move on to other jobs and therefore would have been missed during the sampling process in the present study. On the other hand, those workers remaining may be those who have experienced only mild to moderate levels of disability, which are not enough to warrant leaving or changing the job. Considering the low mean RDQ-24 score in the sample of this study, this is certainly a possibility. Forth, disability due to LBP in different occupations is unlikely to be identical because the physical requirements for different occupations are different. Thus, the association between the religious beliefs and practices of Buddhism and disability could be different among different working populations. Generalization of the results from this study to other populations should be made with caution. Lastly, the present study reported on socially undesirable behavior specifically in terms of the religious beliefs and practices, which may have led to bias. Future studies should consider inclusion of objective information to increase accuracy.

## Conclusions

The current study examined the relationships between the religious beliefs and practices of Buddhism and disability and psychological stress in office workers with chronic LBP. Disability due to LBP was assessed using the Roland-Morris Disability Questionnaire and psychological stress was assessed based on salivary cortisol. We found that the religious beliefs and practices of Buddhism have a significant effect on psychological stress but not disability due to LBP. Chronic LBP office workers with high religiousness experienced lower psychological stress. The findings support the notion that religion/spirituality is associated with positive psychological conditions. We also found that depressive symptoms were associated with both psychological stress and disability status. Thus, depression may be a mediator of the effect of psychological stress on disability in patients with chronic LBP. The findings from this study add to the mounting empirical evidence that the body and mind are inextricably linked and an effective treatment for chronic LBP should incorporate both physical and mental health interventions. However, there is much need for continued research to learn about the complex relationship between religion/spirituality and health.

## Competing interests

The authors declare that there are no conflicts of interest.

## Authors’ contributions

The authors have contributed in the following ways: AS provided concept/research design, data collection, data analysis and manuscript writing. PJ provided concept/research design, data analysis and manuscript writing. TT and PP provided concept/research design and manuscript writing. All authors read and approved the final manuscript.

## Pre-publication history

The pre-publication history for this paper can be accessed here:

http://www.biomedcentral.com/1471-2474/14/29/prepub

## Supplementary Material

Additional file 1 Religious Beliefs and Practices of Buddhism Questionnaire. Click here for file

## References

[B1] Hill JC Fritz JM Psychosocial influences on low back pain, disability, and response to treatment Phys Ther 2011 91 712 721 21451093 10.2522/ptj.20100280

[B2] Manchikanti L Fellows B Singh V Pampati V Correlates of Non-Physiological Behavior in Patients with Chronic Low Back Pain Pain Physician 2003 6 159 166 16883375

[B3] Davis MC Zautra AJ Reich JW Vulnerability to stress among women in chronic pain from fibromyalgia and osteoarthritis Ann Behav Med 2001 23 215 226 11495222 10.1207/S15324796ABM2303_9

[B4] Leeuw M Goossens ME Linton SJ Crombez G Boersma K Vlaeyen JW The fear-avoidance model of musculoskeletal pain: current state of scientific evidence J Behav Med 2007 30 77 94 17180640 10.1007/s10865-006-9085-0

[B5] Pincus T Vogel S Burton AK Santos R Field AP Fear avoidance and prognosis in back pain: a systematic review and synthesis of current evidence Arthritis Rheum 2006 54 3999 4010 17133530 10.1002/art.22273

[B6] Oman D Thoresen CE 'Does religion cause health?' Differing interpretations and diverse meanings J Health Psychol 2002 7 365 380 22112748 10.1177/1359105302007004326

[B7] Baetz M Bowen R Chronic pain and fatigue: Association with religion and spirituality Pain Res Manage 2008 13 383 388 10.1155/2008/263751 PMC2799261 18958309

[B8] Abraodo-Lanza AF Vasquez E Echeverroa SE En las Manos de Dios [in God's Hands]: Religious and other forms of coping among Latinos with arthritis J Consult Clin Psychol 2004 72 91 102 14756618 10.1037/0022-006X.72.1.91 PMC3657202

[B9] Baetz M Griffin R Bowen R Koenig HG Marcoux E The association between spiritual and religious involvement and depressive symptoms in a Canadian population J Nerv Ment Dis 2004 192 818 822 15583502 10.1097/01.nmd.0000146735.73827.85

[B10] The National Statistical Office Statistics in Thailand. [Online] 2009 [cited 2011 Dec 9]; Available from URL: http://service.nso.go.th/nso/thailand/thailand.jsp

[B11] Payutto PA Dhamma Bilingualized 2010 Bangkok: Panya-pawana

[B12] Rosenzweig S Greeson JM Reibel DK Green JS Jasser SA Beasley D Mindfulness-based stress reduction for chronic pain conditions: Variation in treatment outcomes and role of home meditation practice J Psychosom Res 2010 68 29 36 20004298 10.1016/j.jpsychores.2009.03.010

[B13] Cho S Heiby EM McCracken LM Lee SM Moon DE Pain-related anxiety as a mediator of the effects of mindfulness on physical and psychosocial functioning in chronic pain patients in Korea J Pain 2010 11 789 797 20338821 10.1016/j.jpain.2009.12.006

[B14] McCracken LM Gauntlett-Gilbert J Vowles KE The role of mindfulness in a contextual cognitive-behavioral analysis of chronic pain-related suffering and disability Pain 2007 131 63 69 17257755 10.1016/j.pain.2006.12.013

[B15] Schutze R Rees C Preece M Schutze M Low mindfulness predicts pain catastrophizing in a fear-avoidance model of chronic pain Pain 2010 148 120 127 19944534 10.1016/j.pain.2009.10.030

[B16] Kabat-Zinn J Lipworth L Burney R The clinical use of mindfulness meditation for the self-regulation of chronic pain J Behav Med 1985 8 163 190 3897551 10.1007/BF00845519

[B17] Hellhammer DH Wust S Kudielka BM Salivary cortisol as a biomarker in stress research Psychoneuroendocrinology 2009 34 163 171 19095358 10.1016/j.psyneuen.2008.10.026

[B18] Sudhaus S Fricke B Stachon A Schneider S Klein H von Düring M Hasenbring M Salivary cortisol and psychological mechanisms in patients with acute versus chronic low back pain Psychoneuroendocrinology 2009 34 513 522 19028020 10.1016/j.psyneuen.2008.10.011

[B19] IJmker S Blatter BM van der Beek AJ van Mechelen W Bongers PM Prospective research on musculoskeletal disorders in office workers (PROMO): Study protocol BMC Musculoskelet Disord 2006 7 55 16822300 10.1186/1471-2474-7-55 PMC1550718

[B20] Briggs AM Jordan JE Buchbinder R Burnett AF O'Sullivan PB Chua JY Osborne RH Straker LM Health literacy and beliefs among a community cohort with and without chronic low back pain Pain 2010 150 275 283 20603025 10.1016/j.pain.2010.04.031

[B21] Kuorinka I Jonsson B Kilbom A Vinterberg H Biering-Sorensen F Andersson G Jorgensen K Standardised Nordic questionnaires for the analysis of musculoskeletal symptoms Appl Ergon 1987 18 233 237 15676628 10.1016/0003-6870(87)90010-x

[B22] Bhanthumnavin D Vanindananda N Religious belief and practice of Thai Buddhists: Socialization and quality of life 1997 Bangkok: National Research Council

[B23] Waddell G Newton M Henderson I Somerville D Main CJ A Fear-Avoidance Beliefs Questionnaire (FABQ) and the role of fear-avoidance beliefs in chronic low back pain and disability Pain 1993 52 157 168 8455963 10.1016/0304-3959(93)90127-B

[B24] Pensri P Baxter G McDonough S Reliability and internal consistency of the Thai version of Roland-Morris disability questionnaire and Waddell disability Index for back pain patients Chula Med J 2005 49 333 349

[B25] Nilchaikovit T Sukying C Silpakit C Reliability and validity of the Thai version of the General Health Questionaire J Psychiatr Assoc Thailand 1996 41 2 17

[B26] Chida Y Steptoe A Cortisol awakening response and psychosocial factors: a systematic review and meta-analysis Biol Psychol 2009 80 265 278 19022335 10.1016/j.biopsycho.2008.10.004

[B27] Pruessner JC Kirschbaum C Meinlschmid G Hellhammer DH Two formulas for computation of the area under the curve represent measures of total hormone concentration versus time-dependent change Psychoneuroendocrionology 2003 28 916 931 10.1016/s0306-4530(02)00108-7 12892658

[B28] Thoresen CE Spirituality and health: is there a relationship? J Health Psychol 1999 4 291 300 22021598 10.1177/135910539900400314

[B29] Seybold KS Hill PC The Role of Religion and Spirituality in Mental and Physical Health Curr Dir Psychol Sci 2001 10 20 24

[B30] Rippentrop EA Altmaier EM Chen JJ Found EM Keffala VJ The relationship between religion/spirituality and physical health, mental health, and pain in a chronic pain population Pain 2005 116 311 321 15979795 10.1016/j.pain.2005.05.008

[B31] Johnstone B Yoon DP Relationships between the brief multidimensional measure of religiousness/spirituality and health outcomes for a heterogeneous rehabilitation population Rehabil Psychol 2009 54 422 431 19929124 10.1037/a0017758

[B32] Turner JA Franklin G Fulton-Kehoe D Sheppard L Wickizer TM Wu R Gluck JV Egan K Worker recovery expectations and fear-avoidance predict work disability in a population-based workers' compensation back pain sample Spine (Phila Pa 1976) 2006 31 682 689 16540874 10.1097/01.brs.0000202762.88787.af

[B33] Johnston V Souvlis T Jimmieson NL Jull G Associations between individual and workplace risk factors for self-reported neck pain and disability among female office workers Appl Ergon 2008 39 171 182 17761137 10.1016/j.apergo.2007.05.011

[B34] Poleshuck EL Bair MJ Kroenke K Damush TM Tu W Wu J Krebs EE Giles DE Psychosocial stress and anxiety in musculoskeletal pain patients with and without depression Gen Hosp Psychiatry 2009 31 116 122 19269531 10.1016/j.genhosppsych.2008.10.003 PMC2677657

[B35] Woby SR Roach NK Urmston M Watson PJ The relation between cognitive factors and levels of pain and disability in chronic low back pain patients presenting for physiotherapy Eur J Pain 2007 11 869 877 17360202 10.1016/j.ejpain.2007.01.005

[B36] Ruoff GE Depression in the patient with chronic pain J Fam Pract 1996 43 S25 33 8969710

[B37] Hall AM Kamper SJ Maher CG Latimer J Ferreira ML Nicholas MK Symptoms of depression and stress mediate the effect of pain on disability Pain 2011 152 1044 1051 21306826 10.1016/j.pain.2011.01.014

