# Supplementary material for: Are religious beliefs and practices of Buddhism associated with disability and salivary cortisol in office workers with chronic low back pain?
Source: BMC Musculoskelet Disord. 2013 Jan 17;14:29. doi: 10.1186/1471-2474-14-29 (PMC3554421; doi:10.1186/1471-2474-14-29)
Supplement: Additional file 1 — Religious Beliefs and Practices of Buddhism Questionnaire. [file 1471-2474-14-29-S1.doc]

Appendix

**Religious Beliefs and Practices of Buddhism Questionnaire**

**Direction for answer** This questionnaire involves beliefs and practices by Thai people which may vary person from person. Please read the statements below and then consider to what extent these statements are true for you; if “true”, please indicate its degree ranging from “absolutely true”, “true” or “somewhat true” and if “untrue” please indicate its degree ranging from “somewhat untrue”, “untrue” or “absolutely untrue” by ticking ✓ in the relevant box. There are 30 questions in this part and **please answer them all**.

| **Question** | **Absolutely true** | **True** | **Somewhat true** | **Somewhat untrue** | **Untrue** | **Absolutely untrue** |
| --- | --- | --- | --- | --- | --- | --- |
| 1. I believe that following the Lord Buddha’s teachings will make me truly happy. |  |  |  |  |  |  |
| 2. Some monks help me to get more familiarity and faith in a religion. |  |  |  |  |  |  |
| 3. I believe there is no other life after man’s death. |  |  |  |  |  |  |
| 4. The belief that “Life is Suffering” is a pessimistic perspective and nonsense. |  |  |  |  |  |  |
| 5. I believe that merit is the only one thing that I can carry away in another life. |  |  |  |  |  |  |
| 6. I believe that heaven and hell or next life is superstitious. |  |  |  |  |  |  |
| 7. The Lord Buddha is significant to my life. |  |  |  |  |  |  |
| 8. I can be a good man without the need to pay attention to the Lord Buddha’s teachings. |  |  |  |  |  |  |
| 9. Self-development for discovering absolute truth (Nirvana) is not beyond human effort. |  |  |  |  |  |  |
| 10. I appreciate the mercy of the Lord Buddha to mankind. |  |  |  |  |  |  |
| 11. I am willing to tell a lie if it can help me to get benefit. |  |  |  |  |  |  |
| 12. I think prayer before bed is an absurdity. |  |  |  |  |  |  |
| 13. I have to be richer than today first before giving to others. |  |  |  |  |  |  |
| 14. I drink liquor/beer irrespective of that fact that whether such act has breached the commandments. |  |  |  |  |  |  |
| 15. I don’t think meditation can give any benefit to me. |  |  |  |  |  |  |
| 16. Stealing trivial thing from shop should not be an offence as the shop owner can earn a lot of profits from other goods. |  |  |  |  |  |  |
| 17. I like to introduce other person to read the book relating to Buddhism. |  |  |  |  |  |  |
| 18. I am full of spite for whoever deliberately hurts me. |  |  |  |  |  |  |
| 19. Fishing is an exciting sport activity. |  |  |  |  |  |  |
| 20. I always think of sin and its consequences before deciding to do something. |  |  |  |  |  |  |
| 21. I like to visit monastery than any other place. |  |  |  |  |  |  |
| 22. I always think of the Triple Gem (Lord Buddha, Dharma and Monk) when I pass religious place. |  |  |  |  |  |  |
| 23. My career whether at the moment or in the future must not be the one forcing me to violate the commandments. |  |  |  |  |  |  |
| 24. I spend most of time in my life to make good things and merits. |  |  |  |  |  |  |
| 25. I frequently use my spare time to study or practice the Dharma. |  |  |  |  |  |  |
| 26. During hanging out with friends, I usually don’t care whether my act violates any commandment. |  |  |  |  |  |  |
| 27. I used to discuss Dharma issues with other person. |  |  |  |  |  |  |
| 28. I feel that I am just a nominal or registered Buddhist. |  |  |  |  |  |  |
| 29. Religious prepossession can impede the progress in our life. |  |  |  |  |  |  |
| 30. I always help other person and society to make merit. |  |  |  |  |  |  |
